# Environmental Galenics: large-scale fortification of extant microbiomes with engineered bioremediation agents

**DOI:** 10.1098/rstb.2021.0395

**Published:** 2022-08-15

**Authors:** Víctor de Lorenzo

**Affiliations:** Systems Biology Department, Centro Nacional de Biotecnología-CSIC, Campus de Cantoblanco, Madrid 28049, Spain

**Keywords:** synthetic biology, chassis, horizontal gene transfer, *Pseudomonas*, containment, digital twins

## Abstract

Contemporary synthetic biology-based biotechnologies are generating tools and strategies for reprogramming genomes for specific purposes, including improvement and/or creation of microbial processes for tackling climate change. While such activities typically work well at a laboratory or bioreactor scale, the challenge of their extensive delivery to multiple spatio-temporal dimensions has hardly been tackled thus far. This state of affairs creates a research niche for what could be called *Environmental Galenics* (EG), i.e. the science and technology of releasing designed biological agents into deteriorated ecosystems for the sake of their safe and effective recovery. Such endeavour asks not just for an optimal performance of the biological activity at stake, but also the material form and formulation of the agents, their propagation and their interplay with the physico-chemical scenario where they are expected to perform. EG also encompasses adopting available physical carriers of microorganisms and channels of horizontal gene transfer as potential paths for spreading beneficial activities through environmental microbiomes. While some of these propositions may sound unsettling to anti-genetically modified organisms sensitivities, they may also fall under the tag of TINA (there is no alternative) technologies in the cases where a mere reduction of emissions will not help the revitalization of irreversibly lost ecosystems.

This article is part of the theme issue ‘Ecological complexity and the biosphere: the next 30 years’.

## 1. Introduction

**Figure 1.**
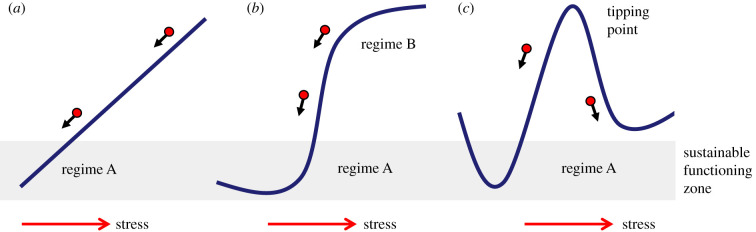
Stability regimes of naturally occurring ecosystems. (*a*) Reversible linear: the system goes through incremental deterioration following increasing stress, but it is reversible along the same trajectory if the insult goes back to the initial level. (*b*) Threshold-dependent, nonlinear behaviour: upon exposure to enough pressure, the system moves from one operational state to a non-functioning regime—but it can return to normal when the burden ceases. (*c*) Nonlinear threshold-dependent with catastrophic fold bifurcation. In this scenario, stress makes the system reach a tipping point followed by irreversibly running into a complete shift of regime [5]. In this case, decreasing the pressure is not enough for returning to the sustainable functioning zone.

The ongoing climate change and the ensuing prospect of a massive extinction is the result of the interplay between the innate geochemical and planetary dynamics of Earth with the impact of anthropogenic activities (in particular industrial/urban emissions and land use) on a large number of otherwise balanced ecosystems [1]. A growing volume of data supports the notions coming from theoretical ecology that human pressure on natural environments often follows a nonlinear threshold-dependent dynamics [2–4] with catastrophic fold bifurcations [5]. Under this scenario, stress may push the corresponding systems to reach a tipping point followed by irreversibly running into the complete shift of regime (figure 1). The literature of the last few years has reported hundreds of instances where such ecological shifts seem to have occurred as a consequence of human action [6]. Once an equilibrium is pushed way beyond a tipping point, simply reducing pressure on them (e.g. by decreasing emissions) will not suffice to return them to a steady functioning. In such cases, proactive, scalable interventions could be a last resort for the restoration of damaged ecosystems of diverse sizes, from local problems to a planet-wide dimension. However, what specific activities should be enhanced to this end and how can they be propagated to a level able to make a difference in such a very large scenario? This question cannot be answered isolated from an understanding of how the whole biosphere works as a complex, dynamic system in which the biological component (including human action) is just one of the various players. A number of activities run by the one species *Homo sapiens*—especially since the industrial revolution, the exploitation of fossil fuels and the onset of intensive agriculture—seems to have hit precisely many of the key nodes that have sustained planetary homeostasis for many millennia. In particular, massive emissions of CO_2_,^1^ methane and other greenhouse gases, e.g. nitrous oxide and a plethora of ozone-depleting fluor-containing chemical species [7]. Although in quantitative terms they are still a very minor portion of the composition of atmospheric gases, even small variations can alter the properties of the protecting shield that enables life on Earth's surface. Such a defence involves not only atmospheric gases with their own protecting ability but also water vapour, the energy involved in state change of which (liquid water to ice and liquid water to vapour) acts as a global buffer of extreme changes of temperatures, leading *inter alia* to formation/dissipation of clouds. Some naturally occurring discharges (e.g. isoprene produced by forests and phytoplankton [8]) may change the shielding role of atmospheric water towards a *cold* direction, while others (CO_2_, CH_4_ and N_2_O) do it into the *warm* course.^2^ For the last millennia, variations of these players could be generally buffered through global, naturally occurring mechanisms of carbon capture in soil, forests and oceans. However, in the last century, human population explosion, industrialization/urbanization, massive farming, intensive agriculture and alteration of land use have changed the contestants that thus far ruled such balances. The main outcome of anthropogenic emissions is their contribution to the well documented increase of the temperature of planet Earth and the dire consequences that it involves if the trend continues unchecked in the same direction [9]. Yet, note that global warming occurs along other large-scale environmental threats, including expansion of drylands [10] and scorched soil, invasion of all types of ecosystems and trophic chains with plastic residues and contamination of food and drinking water with micropollutants and particulates [11]. Air quality, mismanagement of agricultural nitrogen and phosphorus and a mounting volume of lignocellulosic residues also join the list of the tolls of our modern lifestyle [12]. While all of these issues have their own specifics, they are also ultimately connected to our failure to integrate human-created industrial metabolism [13] into the big planet-wide biogeochemical cycles that have operated in the biosphere for a long time [14].

## 2. Mitigation and adaptation: business as usual?

Much of the recent literature and decision-making on climate change deals with mitigation and adaptation to it [15]. In the first case, the efforts aim at decreasing emissions and moving towards a greener industry producing minimal residues and very high recycling levels—what has been called *Circular Economy* [16]. These are welcome developments, but they fail to recognize the dynamics of catastrophic fold bifurcations mentioned above that already afflict many key ecosystems. In these cases, reducing, even altogether stopping the source of stress will not make networks return to the preceding status. In the second instance (adaptation), the edge is about assuming the inevitability of climate change and global warming and take it as an opportunity to maintain customary activities by implementing some changes or even creating fresh business opportunities [17]. The idea in this last case involves stop considering greenhouse gases (and other detrimental chemicals) not as *pollution* to be eliminated but as cheap resources that can be used to make useful products. A number of companies have indeed succeeded in producing high added value items out of CO_2_ captured from air, including, e.g. vodka (https://aircompany.com/products/air-vodka-with-natural-flavors) construction materials (https://carbonupcycling.com), packaging/fashion items (https://expeditionair.today) and even jewellery (https://aetherdiamonds.com). In a more realistic realm, the science and technology of creating value out of C-containing waste—what is being called *carbon upcycling* (CU; [18])—is currently experiencing a considerable push because of its commercial prospects and its considerable marketing potential [19]. In particular, one-carbon biotechnology is indeed one of the frontiers of contemporary research and likely to deliver many marketable items from otherwise wasted precursors [20–25]. These are again welcome, smart developments that make a virtue out of necessity. Unfortunately, the bulk amounts of emissions that can be removed through CU are altogether insufficient to have even a minimal effect in the composition of atmospheric gases and thus very little effect on climate change. Furthermore, most CU processes typically fail to deliver a net balance of C removal—thus often increasing rather than decreasing the problem. In summary, whether mitigation or adaptation, the mainstream discourse thus far on how to deal with a warmer planet focuses on reducing emissions, finding incentives for reshaping/relocating economic activities and converting waste into value in the hope that the same state of affairs can be maintained indefinitely. Alas, these actions—even in the best-case scenario that they are generally implemented—are unlikely to produce the desired effects, specifically on avoiding the critical temperature increase of 2°C which is considered to be a global tipping point in the functioning of the biosphere as we know it [26]. The issue is, therefore, not so much to just moderate anthropogenic impacts but to foster conceptual and material tools for reverting pollution figures to pre-industrial times; but is this technically possible at all?

## 3. The players of global carbon balance

**Figure 2.**
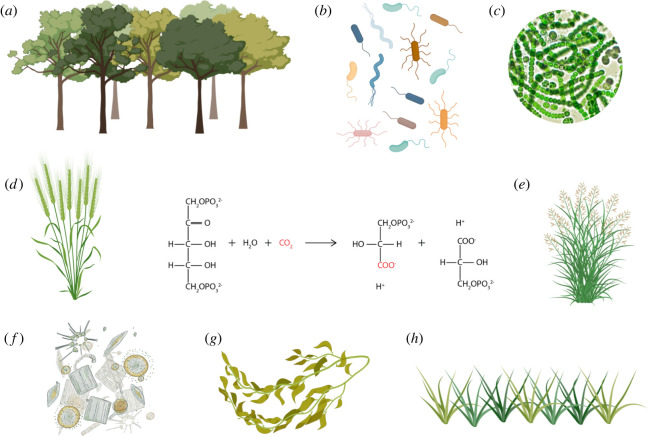
The RuBisCosphere. The enzyme ribulose bisphosphate carboxylase runs what is perhaps the most important reaction of the biosphere by fixing inorganic CO_2_ of the air to the 5-carbon sugar ribulose bisphosphate, generating two 3C phosphoglycerate molecules that can subsequently enter central metabolism. Despite millions of years of evolutionary history RuBisCo is not only very inefficient (fixing only 2–5 CO_2_ molecules s^−1^), but it is also non-specific for CO_2_, and (depending on conditions) it can also add CO_2_ to ribulose bisphosphate and channel the products towards a different metabolic process (photorespiration). Photosynthetic species typically compensate these problems by producing high levels of the protein—it is in fact believed to be the most abundant enzyme on Earth. Various variants of the RuBisCo-dependent Calvin cycle can be found in woody plants (*a*), many types of non-photosynthetic autotrophic bacteria (*b*), cyanobacteria (*c*), crops (*d*), surface greenery (*e*), phytoplankton (*f*), seaweed/kelp (*g*), and grassy plants (*h*). All of these shape the *RuBisCosphere*, the key ruler of the CO_2_ balance in planet Earth.

Existing life in what we call the biosphere is enabled by the interactions among a large number of biotic and abiotic actors forming a densely connected and dynamic network ultimately run by sunlight. Choices for deliberately altering such a network are thus to be found in either the abiotic component or in the biological ingredient. The first scenario is covered by the field of *Geoengineering* [27], an approach that proposes large-scale interventions to modify physico-chemical processes involved in regulating surface temperature, e.g. the albedo, cloud formation, capturing CO_2_ with enhanced rock weathering [28], ocean basification [29], etc. Beyond its technical feasibility, geoengineering is to this day the subject of a considerable controversy [30], including *inter alia* the need of extraordinarily large infrastructures and phenomenal investments for making it happen. This virtually leaves the biological component of the planet's homeostasis as the only other possible actor available to influence large-scale processes. A recent calculation of the different contributors to the whole Earth's biomass [31] indicates that out of a whole of *ca* 545 GTons of live matter, more than 80% consists of plants while the bulk of the rest (approx. 17%) correspond to microorganisms (75% being bacteria), animals adding a mere approximately 0.3% to the total sum. These figures instantly indicate that plants and bacteria are key brokers of the global carbon balance, yet with different roles. Plants (including aquatic species and algae), phytoplankton and many marine bacteria share the ability to fix CO_2_ through different variants of photosynthesis. Depending on the host of the cognate reactions, the molecular species that result from such a process end up in polymers such as cellulose/hemicellulose, lignin, suberin or microbially produced exopolymeric substances, alginate, etc. that act as carbon sinks whether in soil, forests or marine systems. Since the bulk of C capture through photosynthesis ultimately depends on the efficacy of the enzyme ribulose-1,5-bisphosphate carboxylase-oxygenase (RuBisCo), it has been argued that better versions of the same activity could have an immense effect on global CO_2_ balance [32]. RuBisCo is shared by all Calvin cycle-based photosynthetic systems held in the biosphere by a large diversity of biological systems [33], from forests to kelp to cyanobacteria. RuBisCo versions can be found also in different types of autotrophic bacteria and archaea able to fix carbon non-photosynthetically [34]. The merge of all such carriers forms what could be described as a *Rubiscosphere* (figure 2), which is one of the most important supporters (if not the most important) of life on Earth. The enzyme, along with others of the Calvin cycle, has been functionally expressed in non-native hosts such as *Escherichia coli* for enabling them first to recruit CO_2_ into their biomass [35] and then making them virtually autotrophic [36]. This is just one of the various possibilities to artificially enhance CO_2_ processing by non-photosynthetic bacteria [37–39] thereby opening interesting possibilities of increasing carbon retention capabilities of the environmental microbiome through deliberate fortification of their genomic complement with engineered pathways of this sort (see below). In addition, biological approaches coexist and may ultimately converge with purely physico-chemical ones, e.g. electrochemical CO_2_ capture [40].

Given that plants and plant-derived polymers—and their eventual deposition in soil and the sea bottom—are a major destination of photosynthetic carbon, they appear as key instruments for tackling climate change, in particular woody and perennial species. Not surprisingly, massive reforestation/aforestation of land not yet used in agriculture has been proposed as one of the most promising actions for curbing climate change. Bastin *et al.* [41] suggested (not without controversy) that an increase of the tree cover outside of existing forests and agricultural and urban land would have the potential to store an extra equivalent of 25% of the current atmospheric carbon pool—both in the plant biomass and in forest soil. Along the same line, extensive farming of bulky seaweed, e.g. *Sargassum* and other biomass-rich species in the oceans has been entertained—again not without polemic—for capturing large amounts of atmospheric CO_2_ [42] avoiding the problem of land occupation and forest fires.^3^ In either case, the potential of plants and algae (including soil-bound types [43]) could be further increased by engineering specific species for a superior CO_2_ fixation job [44]. Possibilities include not just enhancing the efficacy of the utterly suboptimal RuBisCo [33,45,46], but also by increasing the electron flow through the Calvin Cycle [47], improving CO_2_ internment [48,49], interfacing biological and abiotic systems [50], engineering overproduction of lignocellulosic materials, suberin and other recalcitrant polymers, e.g. sporopollenin [51]. Furthermore, the shape and stress-resistance properties of many vegetal species can be genetically modified to make them amenable to colonize otherwise hostile niches, e.g. saline, afflicted by extreme temperatures, low water, etc. [52]. Current advances in engineering root-associated diazotrophs [53] and the prospects of implementing N2-fixation in plants [54,55] can deliver new species (or their combination thereof) improved in their ability to capture atmospheric carbon into their biomass and expanding the green cover of our planet. Alas, this is not the end of the challenge: much of the vegetal matter can subsequently decay and be degraded by microorganisms which may release CO_2_ back to the atmosphere. A well-settled process to tackle this setback is pyrolysis of biomass into solid carbonaceous products, i.e. biochar, which is growingly considered as one of the few, authentically negative emission technologies [56,57].

With the onset of synthetic biology (SynBio), plants can also be genetically nurtured further for many other properties of environmental interest. Among others, this includes reshaping root architecture [58] for better access to scarce nutrients in poor soils, increasing leaf surfaces for a better exposure to sunlight [59] or avoiding respiratory carbon losses in crops [60,61]. Whether woody or grassy variants, plants can deal also—themselves or along with their root-associated bacteria and mycorrhizae—with a suite of chemical pollutants (e.g. heavy metals, xenobiotics) for soil clean up [62,63]. Finally, note that both plant surfaces and their linked epiphytic bacteria have the potential to remove or degrade chemical pollutants from air and thus help its quality, adding an extra value to their inherent CO_2_ capture abilities [64]. On these bases, it seems evident that both naturally occurring and engineered vegetal species, whether land-based or thriving in marine scenarios should be part of any ambitious strategy to revert climate change, well beyond the traditional objective of increasing crop yields. A considerable bonus of land-based plants as bioremediation agents is the wealth of available expertise on extensive agronomical practices that makes massive sowing and propagation of all types of vegetal species in the soil a largely tractable problem. In comparison, extensive farming of seaweeds for the sake of CO_2_-capturing is still in its infancy, although the endeavour can be helped by the long-time available expertise on kelp farming for the production of edible algae and gelling agents [65].

## 4. The onset of the environmental microbiome

The growing ease of plant engineering thus opens considerable opportunities for developing rationally designed types for efficaciously combatting climate change. However, this generally promising view needs some qualification. Despite the abundance and macroscopic visibility of plants [31], note that if we delete three inert polymers from their composition (e.g. cellulose, hemicellulose, lignin), their contribution, what we could call *biochemically active biomass* of the biosphere comes down to a mere approximately 15% of the whole, while microorganisms take over the leading position—bacteria running with a remarkable approximately 60% of the complete account. This makes the environmental microbiome a pivotal actor in any attempt to modify climate and a clear instrument for any possible intervention to that end [66–69]. Note also that other than fixing and anabolizing CO_2_, the catabolic diversity of plants is relatively limited and their ability to degrade other greenhouse emissions, e.g. methane of fluorinated compounds is null. Fortunately, as indicated above, the other biochemical enabler of Earth's homeostasis (which evolutionarily predated plants) is the whole of the planet's microbiota, i.e. the global environmental microbiome with its amazing connectivity, plasticity and evolvability [67,70]. Furthermore, a suite of microorganisms present in extreme environments (including some phototrophs), can fix CO_2_ through routes alternative to standard RuBisCo-based photosynthesis, e.g. the reductive tricarboxylic acid cycle [71], the reductive acetyl coenzyme A pathway [72] and the 3-hydroxypropionate cycle [73].

The last decade has been one of realization of the microbiome, not single microbial species, as the ultimate performer of everything that matters in the biological world. Because of the variety of locations that microorganisms can occupy, the mobility of species among them and the ease of horizontal gene transfer (HGT, [74]), the microbiome connects all other actors of the biosphere. There is in fact a continuum between physico-chemical, plant and animal niches inhabited by microbes, which form a sort of Ariadne's thread that links all types of biological activities on Earth. Their ubiquity, association to eukaryotic hosts and inter-kingdom HGT abilities [75] produce a flow of signalling and information through virtually all life forms. As the main contributors of biochemical activities and the ones that deliver the bulk of material recycling in our planet, microorganisms are the paramount candidates for bringing climate-related figures to acceptable levels. As a matter of fact, the global environmental microbiome is already accredited for having caused a number of major changes in Earth's functioning along its history (e.g. various anoxic events, H_2_S emissions from the seas, methane poisoning, the great oxygenation event, etc.) and is thus proven to have the capacity to make a difference at such phenomenal scale [76]. Under such a perspective, the global environmental microbiome is the key ally that we have for engineering interventions aimed at combatting climate change and other anthropogenic insults such as methane [77], and other greenhouse emissions, plastics and chemical macro/micropollutants. Finally, the microbial world is the ultimate repository of the metabolic diversity of the biosphere [78] and the one endowed with the maximum capacity to solve complex biochemical problems. The whole environmental microbiome constitutes a sort of planetary brain/gut system, able to process information and return specific solutions to given challenges [79]. The whole of the microbial biomass is thus the one extensive, ubiquitous and genetically determined catalyst that has the capacity to make a difference at the scale necessary to face climate change. In this context, the question is whether it could also be rationally reprogrammed for reverting emissions and other types of pollutants that cause environmental deterioration?

## 5. Engineering synthetic biology agents for bioremediation

The notion of rationally enhancing microbes (so-called genetically engineered microorganisms; GEMs) for extensive biodegradation of toxic waste started to receive considerable attention during the second half of the 1980s and early 1990s [80–82] owing to the availability of effective recombinant DNA technologies, the onset of *green awareness* and the exposure of environmental disasters caused by chemical spills in public media. The early interest on the approach went later through various ups and downs, not only because of regulatory bottlenecks and concerns on safety, but mostly owing to the dearth of sufficient knowledge at that time to identify *in situ* biodegradation bottlenecks. For GEMs to have an effect, the rate-limiting parameter of the process has to be the biology/enzymology. There are many cases where pollutants could not be degraded by natural communities because the available degraders were scarce or poorly active. In such circumstances, the addition of a designer GEM to the scenario vastly accelerated biodegradation [83], e.g. by protecting the entire food web of the ecosystem. However, in other settings ruled by different rate-limiting parameters, the addition of GEMs failed to deliver catabolic activities to afflicted sites in a fashion superior to naturally occurring microorganisms [84,85]. While many environmentally interesting activities were genetically knocked-in in bacteria growing in a Petri dish or in a bioreactor, they generally turned useless when inoculated in a natural scenario. GE-driven bioremediation has since kept a low profile until the arrival one decade later of systems and synthetic biology [86,87]. These new conceptual and material frames are re-empowering the field for addressing environmental pollution problems that were considered intractable before, including reversal of greenhouse gases emissions, elimination of plastics and micropollutants as well as other large-scale issues [12,88–90]. The scenario that enables this renewed opportunity includes: (i) a much better understanding of microbial ecology at multiple scales and the interplay of environmental bacteria with their surrounding physico-chemical settings; (ii) the realization of the microbiome and the microbial consortia as the factual performers of biological activities previously attributed to isolated strains; and (iii) the wealth of molecular and computational tools available for designing, testing and optimizing genetic constructs. Moreover, machine learning (ML) and advanced modelling [91–93] now offers a much safer ground for guiding and predicting the fate and effects of releasing SynBio agents (SBAs) to different environments and scales [94,95].

**Figure 3.**
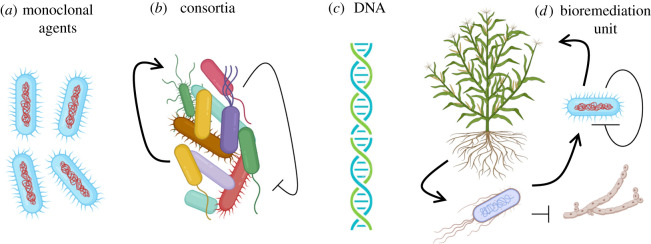
Biological agents for environmental bioremediation. Pathways for mitigating and even reversing the effect of emissions on different types of ecosystems can be channelled to the target sites in various biological formats. The simplest is having the trait(s) encoded in the genome of a monoclonal strain (*a*) that can be delivered to the location of interest. Alternatively, the same and more of such traits can be embodied in an engineered consortium (*b*) with a predefined division of labour and positive negative interactions between components of the partnership designed for the sake of robustness. Yet, as discussed in the text, colonization resistance may make direct inoculation inefficient, in which case propagation of DNA (*c*) with sequence features for expression of the cognate genes in a variety of hosts through HGT can be considered. Finally, a complete, self-sustained bioremediation unit (*d*) can be assembled by combining components recruited from various biological domains and endowed with a predefined relational logic and niche-creating abilities—what has been called *Terraforning motifs*.

**Figure 4.**
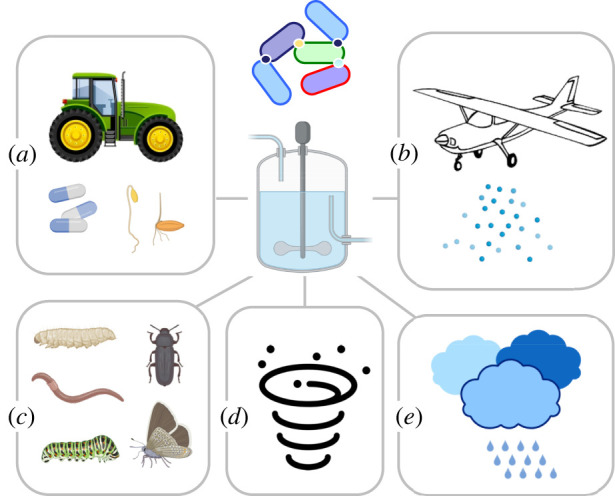
Some possible strategies for large-scale propagation of bioremediation biologicals. Once a given self-propagating biological is generated in the laboratory and produced on a small scale it needs to be formulated and delivered extensively to the target sites. Various possible avenues to this end are sketched. (*a*) Coating of plant seeds or preparation in seed-size biodegradable capsules and dispersion in soil with agronomic machinery. (*b*) Spreading onto soil or aquatic systems with aerosols or aerial sprinkling of aqueous suspensions. (*c*) Portage in animal (e.g. insects, worms) carriers. Insects in various development stages are excellent transporters of microorganisms while worms are major soil processors, e.g. in composting. There are ample opportunities for combining the power of some species or their larvae to grind solid pollutants (e.g. plastics, lignin residues) with fortification of their microbiome for their biodegradation. (*d*) Wind, tornados and dust storms take solid particles and their associated microorganisms to higher layers of the atmosphere through which they can be transported at very long distances. This offers a highway for long-range circulation of biologicals of all types. (*e*) Clouds are excellent natural niches for methanotrophs and many other types of bacteria and can travel to far locations. Cloud microbiology could converge with artificial cloud seeding for area-wide release of biodegradation agents to a large variety of places.

These developments have occurred at a moment when the focus of pollution has moved from discrete locations (e.g. petroleum or chemical spills in given sites) to large-scale, even planet-wide scenarios. The time has thus never been so ripe to look at the global environmental microbiome as the key ally that we have for sound planning of interventions aimed at combatting climate change and other anthropogenic insults. From a practical point of view, this issue has three somewhat separate technical challenges: (i) design and implementation of bioremediation reactions including—but not limited to— those leading to a net outcome of C capture; (ii) effective expression of such engineered pathways in suitable hosts or consortia thereof (figure 3); and (iii) large-scale propagation of the improved agents and activities through the environmental microbiome (figure 4).

The first question, i.e. engineering bioremediation reactions, is largely the mission of contemporary synthetic biology and metabolic engineering [87]. The field has witnessed in the last few years an explosion of genetic constructs encoding activities of environmental interest in the context of climate change and ecosystem deterioration. Apart from a plethora of propositions for manufacturing chemicals through environmentally friendly bioprocesses [96,97], contemporary SynBio has paid much attention to microbial activities relevant to global environmental sustainability. Photosynthesis rests on top of these as the most important process of C fixation and O_2_ regeneration in the biosphere. As indicated above, regardless of the specific host—and nutrients N, P notwithstanding—the enzyme RuBisCo turns out to catalyse the most critical biochemical step that limits the whole process. It has been recently calculated that even a marginal increase in RuBisCo activity, if propagated at large scale, would have dramatic consequences for the planet's climate [32]. On this basis, it is no surprise that many current efforts are directed to this end, although the very reaction mechanism makes this task very difficult. In turn, this has triggered the exploration of entirely artificial and very efficient CO_2_-fixation pathways [46,98–100] based on the refactoring of O_2_-tolerant carboxylases. While some of such engineered processes work well *in vitro*, they have not yet been fully implemented *in vivo,* perhaps owing to the need of high reductive power in the rate-limiting carboxylation step.

A separate but key angle of the carbon catch is the equation photosynthesis versus respiration. Because about 30–60% of the carbon plants fix is respired by the plants themselves, and plant respiration, planet-wide, is of the same order as anthropogenic CO_2_ production, there is clearly a reason to think not just about increasing CO_2_ fixation but about cutting respiration too [60,101,102]. Apart from CO_2_ balance, current SynBio also aims at developing pathways and strategies for increasing metabolization of methane [103] and decreasing its emission [77], improving the biological reduction of NO/N_2_O [104] and biodegrading fluorinated gases [105]. As indicated above many of such endeavours (in particular CO_2_ management) are currently more directed to generate value out of waste than the mere removal of the target molecule [106]. Still, one-carbon biotechnologies open good avenues for tackling environmental CO_2_ balance in earnest [20].

A separate whole of contemporary SynBio efforts focuses on biodegradation or upcycling of polymers (in particular plastic residues [107,108]), toxic chemicals, micropollutants and lignocellulosic waste [109]. Finally, biological N fixation, recovery of soluble P and capture of water in dry soil have received considerable attention as problems that can be addressed with SynBio tools [12]. Note that in many cases such genetic devices are implemented in model bacteria (e.g. typically *E. coli*) and shown to work as a proof of concept in the small scale and controlled conditions of the laboratory. The obvious next question is how to deliver the same beneficial activities at very large scale in an efficacious, safe and predictable fashion. This touches upon the other two challenges mentioned above: effective expression of the engineered traits in optimal biological carriers and large-scale propagation of the resulting agents through the environmental microbiome. The subject is somewhat reminiscent of the issue of entering an active principle of therapeutic value into a sick body [110]. As argued below—there is much to learn from the roadmap that a new medication, treatment or vaccine have to follow from initial discovery to full-fledged application to actual patients.

## 6. Environmental Galenics

Within traditional pharmacology, the subject known as *Galenics* or *Galenic Science* is the branch responsible for the preparation of drugs and active ingredients as materials that are easy to administer and that provide an adequate therapeutic response [111]. To this end, the efforts focus on the physical carriers of the active principles and the conditions that allow maximum safety and efficacy. Galenics depends on pharmacokinetics and pharmacodynamics to assess the effect of the body on the drug and the drug on the body, respectively. It is easy to pose a direct equivalence between these notions and the scenario of environmental remediation with engineered agents discussed in the paragraphs above. In this context, the term *Environmental Galenics* (EG) could describe the science and technology of releasing designed biological agents into deteriorated ecosystems for the sake of their safe and effective recovery. In this context, the idea boils down to (i) identify the best possible carrier of an improved, genetically encoded trait in terms of expression when and where needed, (ii) material formulation and massive proliferation of the thereby optimized agent, and (iii) monitoring the environmental fate and effects of the engineered biological catalysts.

The first point at issue when entertaining environmental release is the choice of what in SynBio jargon is named the *chassis* [112–115]. Note that it is not enough for an environmental microorganism to act as a host of recombinant DNA to become a *bona fide* chassis for the type of application discussed here. The microbial host at stake must also fulfil a large number of requirements [113,116]. Some species and strains such as the soil bacterium *Pseudomonas putida* [117,118] and its derivatives have emerged as a prototype of bioremediation agent in soil and a testbed for inspecting the many questions regarding the release of SBAs. Many other chassis have been proposed and are being actively developed for application in different types of target ecosystems [114]. Unlike performance in a bioreactor, entering new activities in an open environment asks for additional issues beyond biochemical performance. Besides stress resistance, a critical one is the stoichiometry and the biochemical background for each of the steps involved in a pathway. In many cases this advises adoption of multi-strain and multi-species communities instead of focusing on single specimens. These assemblies can quickly adapt to specific conditions by changing their relative composition instead of regulating gene expression at individual levels [119–121]. Apart from combining biochemical capacities, the components of a microbial partnership can be physically connected by means of surface-exposed adhesins that follow certain association rules [122] and programmed to adapt what has been called *synthetic morphologies* [123,124]. Taken together, playing with physical morphologies enables upgrading of catalytic performance beyond the mere expression of a given pathway. Note that notion of SynBio chassis has been thus far applied mostly to microorganisms, but there is a whole range of potential biological carriers of engineered and environmentally useful activities, e.g. plants (including seaweeds) and animals, in particular nematodes and insects. These can by themselves or in combination with others be key components of biological formulations for tackling emissions and other pollutants (figure 4).

Whether single strains or consortia, the ensuing step involves their preparation in a physical format amenable to extensive dissemination. This challenge is not without precedent, as even traditional agricultural practices have incorporated crop-protecting microbes in their extensive seed-sowing protocols [125,126]. Manufacturing environmental agents in water-soluble capsules with formats and sizes not very different from commercial seeds can indeed facilitate their dispersion [127,128]. Furthermore, encapsulation enables combining the live biological component with additives to increase its catalytic performance and their safety. For instance, capsules can be formulated with osmoprotectors to prevent lethal desiccation [128], safeners to improve efficacy and added nutrients for maintaining biological activity under oligotrophic conditions. The formulation can also be designed for the sake of containment by making the viability of the active agent dependent on one of its ingredients, e.g. auxotrophy for a natural or synthetic amino acid or metabolite added to the capsule or any other physical carrier [129,130]. Foams, plastic tubing and straight spreading of aerosols bearing catalytic agents have also been entertained as strategies for area-wide dispersal of bioremediation agents. These stratagems may be successful for inoculating soil and plant microbiomes with transient or permanent new members. However, there is still much to develop for effective delivering of engineered microbial agents to larger spaces, let alone vast marine regions (figure 4).

One appealing vehicle to this end is microbial propagation and long-distance circulation in clouds. A large number of microorganisms (including methanotrophs [131]) thrive suspended in the aqueous aerosols of clouds [132,133] and can move and be deposited at long distances [134]. On this basis cloud simulation/modelling [135–137] and bioengineering could eventually converge with their artificial generation with already available technologies, e.g. cloud seeding [138]. Along the same line, the Earth's atmosphere enables an intercontinental flow of dust particles that carry a large variety of bacteria, viruses and fungi [139,140]. Since such highways for massive transport of microorganisms are already there, it might be possible to exploit them for the sake of disseminating bioremediation agents to locations that might be difficult to access otherwise. By having a repertoire of activities, possible hosts/chassis material shapes for their large-scale manufacturing and propagation channels, the field is then ready for tackling higher-order ecological engineering. This may be designed with diverse components for autonomous performance as creators or new niches and/or fortification/rehabilitation of existing ones. The specific architecture of such systems needs to be determined on a case-by-case basis, but the constellation of necessary activities and the interplay among them can be modelled in advance with what have been called *Terraforming motifs* [141] and, in a superior scale, *ecological hypercycles* [94]. These concepts and their underlying conceptual frames are beyond the scope of this article, but they are the necessary theoretical counterparts for making large-scale SynBio-based bioremediation a viable scheme.

## 7. Engineering horizontal transfer of beneficial traits

Promising as it may look at first sight, the direct spreading of biological agents for the delivery of novel activities is not devoid of bottlenecks [142]. Ecological theory is well aware of what has been called *colonization resistance* (CR), i.e. the difficulty to stably incorporate a new member in well-balanced community. This was in fact one of the main obstacles for releasing biodegradative GEMs in the first wave of attempts in the late 1980s and early 1990s mentioned above [85]. There are many mechanisms involved in CR, but the final typical outcome is the quick loss of efficacy and eventual dying away of the engineered agent. A frequent setback is that the transient advantage of the engineered strain can result in its overgrowth which in turn creates a niche for predatory protists [143,144].

Although SynBio approaches enable counteracting some of the issues implicated (in particular genetic stability), CR still remains a major obstacle for the implementation of the strategies discussed above, unless there is a special situation regarding the pollutant that gives a clear selective advantage to the biodegradative GEM. Fortunately, there is a complementary line of attack for the wide-ranging dispersal of valuable biological properties: HGT [145]. In this case, the issue is not so much colonization of a standing niche by a new member but the acquisition of a new capacity by an existing microbiome through the deliberate provision of the cognate genetic information. Under this perspective, the repertoire of biological agents that can be engineered includes not only specialized strains and communities of the type discussed above but also spreaders of DNA sequences bearing beneficial properties by means of programmed HGT. Obviously, the technical question of how to disseminate live agents and/or fostering transmission of selected genes at a very high level turns around the traditional pursuit of biological and genetic containment^4^ for a somewhat opposite quest of securing persistence and promoting DNA propagation [129,146].

Domesticating HGT for DNA-based fortification of microbiomes (and complex niches at large) involves at least three fresh research challenges. The first is the development of super-promiscuous gene transfer platforms along with the elimination of HGT barriers. Textbooks traditionally list three major separate mechanisms for gene mobility: conjugation, transformation and transduction. More recent literature, however, tends to tear down the barriers between the three, as accredited by observations on integrative conjugative and mobilizable elements [147–149], lateral transduction phenomena [150], and the interplay between natural transformation and parasitic mobile genetic elements [151]. Yet, the classical conjugal transfer is the one that looks more promising in the shorter term for developing schemes aimed at passing DNA among a wide variety of possible recipients. This is because conjugation naturally occurs among a considerable diversity of interspecific, trans-specific and even trans-kingdom partners [74,75]. In addition, there is already a wealth of information of the global-scale conjugal transfer of genes stemming from studies on the dispersal of antibiotic resistance [152]. While naked nucleic acids and phages are also important players in global genetic traffic (including metabolic and biodegradative genes [153]), their narrower range of acceptors limit their potential for promiscuous DNA spreading. By contrast, out of the many plasmid types, the ones that belong to the incompatibility group IncP1 encode a transfer machinery that seems to be extraordinarily unrestrained regarding the type of possible recipients [154]. They are, therefore, excellent candidates for designing highly efficient DNA transfer platforms among members of a given microbiome and beyond. Such transfer events are expected to occur in communities endowed with different mechanisms not only of CR but also barriers to non-self DNA, e.g. restriction systems, CRISPR (or similar) devices, Type VI secretion of toxins, etc. Designing highly promiscuous carriers of novel DNA thus asks for counteracting most of these barriers for an efficient community-wide spreading of the genes of interest (GoI). This is not out of reach, as increasing knowledge of each such mechanism enables possibilities for overcoming them for the sake of increasing conjugation rates. Once a new DNA sequence enters a target host, it may remain as an extrachromosomal replicon (which may then continue its conjugation course through other members of the microbiome) or integrate in the genome through either recombination or transposition (again, amenable to genetic programming).

However, making DNA to promiscuously enter new hosts is not the end of the story, as the GoI have to be duly transcribed and translated in the physiological and genetic contexts of the receiving cells. A second challenge is, therefore, the host-independent expression of such GoIs. Owing to the conservation of the genetic code, structural sequences of most genes are encoded by equivalent DNA segments; but signals for their transcription, translation and regulation thereof, let alone their interplay with the gene expression machinery, do change dramatically depending on the specific host. This raises an interesting question on the design of universal expression systems for securing activity of the GoIs in any biological host—or using SynBio jargon, engineering *orthogonal heterologous expression* [155]. Transcription and translation signals of promiscuous mobile elements look like a good source of parts for engineering equally unrestrained expression devices [156]. Along the line, platforms based on viral promoters and polymerases have been proposed to this end [157]. However, in either case, there is still considerable room for improvement. The issue of predictable expression is not alien to that of genetic stability and DNA sequence preservation along time, a concern shared with other branches of contemporary biotechnology. As before, some stratagems have been implemented to mitigate mutations in recombinant DNA constructs [158–160] but there is still a wealth of stimulating fundamental questions to answer until the matter can be managed in a large environmental context.

The remaining major challenge for engineering large-scale HGT is that of selective pressure for bringing about the dispersal of GoIs. Standard evolutionary theory would tell us that such a propagation will probably succeed when the acquisition of DNA results in a competitive survival/growth (i.e. fitness) advantage in respect to others. This scenario is clearly substantiated with massive data on global spreading of antibiotic resistance genes. Typically, a new antimicrobial drug starts to be used at a given time and a few decades (if not just years) later cognate genes for resistance to it are found in microorganisms all over the planet [161]. While this accredits the efficacy of the natural HGT mechanisms for circulating new DNA sequences though all types of ecosystems, it also exposes the need for a strong external coercion for such a transmission to happen. Some environmentally beneficial activities (e.g. improving CO_2_ capture, N_2_ acquisition, methane metabolism, plastic digestion, etc.) are expected to result in fitness gains for those hosts that acquire the corresponding new capacities. However, others (e.g. degradation of micropollutants) might be more dubious in that respect. At least two schemes have been put forward to tackle this. In one case, the idea is to intermingle the sequence of the GoI with that of an essential gene or one that gives a clear fitness advantage [158]. In this way, the inconspicuous gene sort of *piggybacks* on the one that enables endurance to an external demand. A second scheme involves the implementation of bacterial versions of *gene drives* (GD). In diploid organisms, GDs favour inheritance of DNA sequence over another regardless of the function it encodes through Cas9/gRNA-mediated cleavage of one of the chromosomes and copy of the cargo sequences via homology-directed repair onto the other chromosome. A prokaryotic variant has been proposed [162] that copies a gRNA cassette and adjacent cargo flanked with sequences homologous to the targeted gRNA/Cas9 cleavage site. These and other strategies to be developed will be invaluable for pursuing the DNA-spreading objectives enunciated in this article.

## 8. Monitoring efficacy and securing the safety of engineered biological catalysts

All the views and technical choices for large-scale bioremediation discussed above are entertained in a time of public concern about the safety, security and unintended consequences of genetic engineering and SynBio [163]. Does it make sense to think on the massive release of GE and SynBio agents for impacting climate given the controversies and legal bottlenecks surrounding comparatively minor interventions like planting GM crops or farming transgenic animals? This article advocates an affirmative response on the basis of the following. First, the genetic technologies available for constructing in the laboratory safe and affective biological devices have improved various orders of magnitude in the last decade and public acceptance of successful constructs able to solve problems has increased likewise. The success of RNA-based vaccines to combat the COVID-19 epidemics bear witness of how an embryonic technology that was thought to remain as a laboratory tool for many years becomes in no time a key instrument to check a global problem [164]. Technical competence in handling recombinant DNA has gone hand by hand with a wealth of ecological knowledge and robust mathematic and computational tools for large-scale modelling of that which were not at hand before [32,94]. Second, the uncertainties on the impact of releasing agents or circulating new genes through microbiomes and other biological landscapes [152] can be tackled through stepwise monitoring of their behaviour at many different scales and environmental conditions. This should provide an affluence of data amenable to ML and other analysis tools which in turn can guide specific interventions and predict their outcome. Finally, as many vital ecosystems are being pushed well beyond a tipping point [6] somewhat meek measures such as reduction of emissions will not suffice to solve the larger problem. In fact, large-scale interventions of the sort entertained here could be considered TINA (there is no alternative) choices for procuring the continuity of Earth as a habitable planet. Yet, these considerations do not hide the necessity to develop robust methods to assess the performance and safety of whatever biological we wish to place in the environment. As indicated above, there is much to learn from the medical and pharmaceutical practices. How could this work?

**Figure 5.**
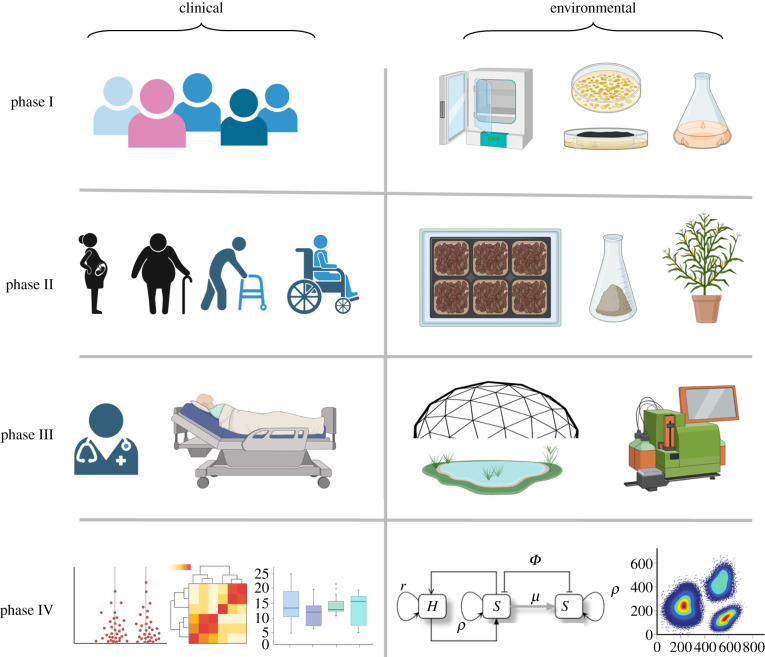
Comparison of standardized phases of clinical trials that precede approval of a new drug or treatment versus possible stages of testing efficacy and safety of biologicals for environmental release. In the human health-related roadmap, phase I studies application to a small group of people just to evaluate safety and possible side effects. Phase II studies both the efficacy and possible adverse effects of the treatment in a larger group of more diverse individuals. The critical phase III extends efficacy/safety studies to still larger population of patients in different regions and countries. If the outcome is positive, it may suffice to be approved. Phase IV is a post-adoption surveillance stage that studies effects that were not seen in earlier trials. Equivalent stages for environmental applications could involve the following. Phase I, testing the biological in laboratory-scale microcosm with a limited number of components for stability, expression of the trait of interest and impact on structure of a pre-existing community. Phase II, same in mesocosms with more actors and a larger variety of physico-chemical conditions. Phase III, scaling-up to an ecotron-size with a complete ecosystem including soil and water matrices along with plants, insects and a controlled, contained atmosphere—along with instruments to quantify relevant parameters. Phase IV involves post-adoption analyses and modelling of the new scenario and surveillance of emergent phenomena.

The well-standardized roadmap that enables the journey from discovery of a new active principle to a full-fledged application to patients involves four subsequent phases of clinical trials (figure 5). These concepts of stepwise clinical trials can be sensibly translated to risk/efficacy assessment of advanced environmental remedies based on innovative GE and SynBio biologicals. Once the ecological parameters and thresholds of interest are chosen it is certainly straightforward to entertain an equivalent roadmap from the laboratory to large scale application. Model ecosystems of increasing complexity and a growing number of components are already available from the size of a microcosm in a Petri dish to a mesocosm and then to ecotrons [165,166] able to reproduce complex ecosystems in a contained space composed of all microbial, plant, water and atmospheric actors. Available technology also permits miniaturization (and therefore multiplexing) of habitats where testing such parameters could be done in a high-throughput fashion. Taken together, these approaches can produce the tools necessary for large-scale rational ecosystem design, a challenge not devoid of precedents [167,168].

One traditional concern regarding the release of engineered agents into the environment is preventing the escape of the engineered strains from the location they were expected to perform and/or dispersal of recombinant DNA to other hosts [163]. This matter has fostered a large number of sophisticated propositions for both biological containment and genetic containment [129,146,169]. True, some of them might be close to achieve what has been called *certainty of containment*. However, also true, that for the type of large-scale application pursued in this article, the objective is not genetic containment but exactly the opposite: massive ecological promiscuity of recombinant genes encoding beneficial activities. Note that permanent takeover of pre-established bacterial communities by an artificially introduced member is unlikely in any case not only because of the CR mechanisms discussed above but also for the intrinsic ecological dynamics of biological consortia. Obviously, the type of HGT advocated here asks for easing rather than restricting the gene flow. In this case, the realistic theme is not containment but tracking both the agents along their DNA and tracing them to a specific origin or designer. Methods for barcoding, tagging and watermarking strains and synthetic constructs based on unique DNA identifiers have been put in place in the last few years [170–173] that facilitates such a task that even permits access to *digital twins* of the biologicals at stake.

## 9. Conclusion and outlook

Compared to the interest of modern biotechnology in tackling human health problems, the attention payed by molecular biology to climate change and ways to crack it has been relatively marginal. A recent (and very conservative) report on the research and development (R&D) spending to bring just one single cancer drug to the market in 2017 indicated an average of $648.0 million per molecule, with median times to develop them of 7.3 years [174]. The same or even higher figures apply to advanced treatments of personalized medicine, let alone gene therapies, etc. Typically also, the revenues of such expenditure within the next 4 years average close to 10 times the initial venture. A seminal academic discovery with potential relevance to human health (e.g. CRISPR) used to be quickly passed to the commercial sector to manage successive rounds of investment for eventual production of marketable drugs and therapies with very high added value. This scenario creates an optimal convergence of the technology push with the market pull, which reaches out the setting of fundamental research priorities. Alas, this is not the case with R&D in environmental research and remediation, in which public interest has not been translated directly thus far to either massive academic attention or market demand. For the time being the environmental market, including that for C capture, is nearly exclusively driven by legislation, which is often watered-down by short-term political and national interests. The challenge to make advanced large-scale environmental interventions a reality involves turning climate change into an opportunity for the creation of wealth (https://medium.com/@tbaruch/the-synthetic-biology-climate-change-opportunity-af8af04d9d5d). To this end, strengthening translational research in the field is badly needed. This article advocates EG, specifically microbiome fortification and activity propagation as key instruments to this end. However, obviously what we call *the environment* is a quite complex system (albeit probably not more than a human body) that requires transdisciplinary approaches encompassing disciplines that have so far remained separate. For instance, it is shocking that models of C capture by forests tend to ignore soil microorganisms as active agents in the process [175]. This is just but one example that exposes the fractionation that environmental studies still endure. Also, there are numerous question marks on the consequences of placing engineered agents in existing ecosystems, both regarding long-term evolutionary consequences of stimulating HGT as well as efficacy proper. This last issue largely depends on having more accurate metrics of CO_2_ traffic, energy consumption and actual yields of the improved activities discussed above.

Mankind is expected to consume 560 Exajoules of energy by 2050, approximately 80% of which is of fossil origin [176]. Given that the existing green cover of the planet captures at best 0.5 W m^−^^2^, it will not be straightforward to durably replace the ongoing consumption of the accumulated carbon capital by a renewable alternative: the whole surface of the Earth would probably not be enough to *naturally* seize so much energy from the Sun. Advanced methods to develop realistic and effective systems to handle these demands are thus badly needed [177]; but this cannot happen without profound changes in the way we understand and implement progress and development in the social and political sphere. Whatever biology can do to prevent/reverse catastrophic developments, it cannot succeed without stopping current tendencies—some of which may not rest without internationally binding legislation and a vigorous implementation. The reality is that if there is money-power to be gained by environmental misdeeds, they will continue, irrespective of the consequences for planetary wellbeing [110]. However there are reasons for optimism: similarly to what happened to health-related biotechnology (and given adequate levels of funding), it should be technically possible to tackle one by one each of the problematic issues mentioned above and deliver safe, efficacious tools for interventions of the sort entertained in this article.

## Data Availability

This article has no additional data.

## Footnotes

1 Regular updates on carbon emissions can be followed in https://www.carbonbrief.org/state-of-the-climate-how-the-world-warmed-in-2021.

2 For a thorough resource on the status of these issues see https://www.acs.org/content/acs/en/climatescience/about.html.

3 For a thorough review of possible strategies of C capture in the oceans see http://nap.edu/26278.

4 For a compilation of containment strategies see https://standardsinsynbio.eu/biocontainment-finder/.
